# Amplitude spectrum distance: measuring the global shape divergence of protein fragments

**DOI:** 10.1186/s12859-015-0693-y

**Published:** 2015-08-14

**Authors:** Clovis Galiez, François Coste

**Affiliations:** Inria Rennes - Bretagne Atlantique, Rennes, France

**Keywords:** Protein, Structural comparison, Fourier transform, Pseudometric, Insertions and deletions

## Abstract

**Background:**

In structural bioinformatics, there is an increasing interest in identifying and understanding the evolution of local protein structures regarded as key structural or functional protein building blocks. A central need is then to compare these, possibly short, fragments by measuring efficiently and accurately their (dis)similarity. Progress towards this goal has given rise to scores enabling to assess the strong similarity of fragments. Yet, there is still a lack of more progressive scores, with meaningful intermediate values, for the comparison, retrieval or clustering of distantly related fragments.

**Results:**

We introduce here the Amplitude Spectrum Distance (ASD), a novel way of comparing protein fragments based on the discrete Fourier transform of their *C*_*α*_ distance matrix. Defined as the distance between their amplitude spectra, ASD can be computed efficiently and provides a parameter-free measure of the global shape dissimilarity of two fragments. ASD inherits from nice theoretical properties, making it tolerant to shifts, insertions, deletions, circular permutations or sequence reversals while satisfying the triangle inequality. The practical interest of ASD with respect to RMSD, RMSD_d_, BC and TM scores is illustrated through zinc finger retrieval experiments and concrete structure examples. The benefits of ASD are also illustrated by two additional clustering experiments: domain linkers fragments and complementarity-determining regions of antibodies.

**Conclusions:**

Taking advantage of the Fourier transform to compare fragments at a global shape level, ASD is an objective and progressive measure taking into account the whole fragments. Its practical computation time and its properties make ASD particularly relevant for applications requiring meaningful measures on distantly related protein fragments, such as similar fragments retrieval asking for high recalls as shown in the experiments, or for any application taking also advantage of triangle inequality, such as fragments clustering.

ASD program and source code are freely available at: http://www.irisa.fr/dyliss/public/ASD/.

**Electronic supplementary material:**

The online version of this article (doi:10.1186/s12859-015-0693-y) contains supplementary material, which is available to authorized users.

## Background

Evaluation of the structural similarity of two proteins is an important task in bioinformatics that is mainly performed at three levels: global protein comparison, structural motif comparison (for spatially contiguous pieces of structure) and fragment comparison (for sequentially contiguous pieces of structures).

We focus here on scoring the similarity of fragments, a task receiving an increasing interest since it is a practical cornerstone for:

- Mining fragments related to a particular protein function [1];
- Building global structural alignment by combinatorial extension [2];
- Representing globally a structure [1] and comparing globally two proteins as a bag-of-fragment of variable length [3], or fixed length [4];
- Comparing/clustering fragments in order to feed learning algorithms to infer structural alphabets/building blocks for protein structure prediction [5–8];
- Assessing the structure prediction from sequence by comparing locally predicted fragments with their native conformation [9].

The classical score used to measure the dissimilarity of two protein structures is the coordinate *root-mean-square deviation* (RMSD) defined as the minimum average distance between superimposed atoms (usually the *C*_*α*_) of the proteins by optimal rigid-body rotation and translation. Drawbacks of RMSD are well known: it necessitates computing the optimal superimposition of the atoms, it tends to increase with proteins’ length and it is more sensitive to local than global structural deviations. Many other measures have been proposed [10], and among those, one has to cite the *distance* variant of RMSD, the RMSD _*d*_ [11]. Rather than comparing the 3D coordinates of the atoms, it performs a more global comparison of the internal distance matrices of each protein, alleviating this way the need of superimposing the structures thanks to the invariance of internal distances by rotation and translation (at the price of not distinguishing mirrored structures). More recently, an interesting advance in measuring the similarity of protein fragments has come up with the Binet-Cauchy (BC) score putting forward several advantages over RMSD: it avoids explicit structure superimposition, enables mining mirror image fragments, is less sensitive to fragment lengths and provides better discrimination of medium range RMSD values [12]. BC score, RMSD and RMSD _*d*_, are computable by tractable *exact* algorithms. Moreover, they do not rely on expert-chosen parameters, so that they universally apply for protein fragments. The limitation of these scores is that they measure the distance between two ordered sets of residues already aligned one-to-one (the *i* ^*th*^ residue of the first set is aligned with the *i* ^*th*^ residue of the second set, typically in the same order than in the fragments’ sequences), making them less suited for the comparison of homologous fragments with mismatches resulting, for instance, from insertions or deletions.

In order to cope with mismatches, an approach is to search for the best (sub)alignment between the residues of both fragments. The problem is then to conciliate two conflicting goals: maximize the number of aligned residues and minimize their structural deviation. A way to quantify the best practical trade-off has been designing scores normalized with respect to the alignment length relatively to their expectation between random proteins. This includes well-known scores developed for the comparison of whole protein structures such as the TM-score used in TM-align, and its successor Fr-TM-align, weighting the close atom pairs stronger than the distant matches to focus more on global fold than local variations [13, 14], or the Z-score of DALI based on a measure of the relative dissimilarity of the distance matrices, weighting down the contribution of pairs in the long distance range by an exponential envelop function [15]. While useful in practice, these scores rely on underlying models of typical random structures and are thus biased by construction towards particular application domains, as witnessed by the presence of “magic numbers” in their formula. Another issue is that the problem of finding the best alignment optimizing these scores is usually difficult and programs such as TM-align, Fr-TM-align and DALI rely thus on heuristic methods that do not guarantee that the optimal score has been found. A remarkable exception is DALIX [16] which introduces an exact and worse-case exponential algorithm that can already be used to align optimally some protein domains in reasonable time with respect to DALI’s objective function. Finally, let us remark that in the best (sub)alignment approach, unmatched residues do not contribute to the overall score. Scoring of the alignment deals with the aligned parts of the structure but no matter how the structures look like over the non aligned part, the score will remain the same. This can be a critical issue for many tasks. For example, when clustering protein fragments, if the similarity of fragments is assessed only over the aligned part then it will lead to inconsistent clusters: a fragment A may be identical to a fragment B over its first 70 % of structure, a fragment C can be identical to the same fragment B over its last 70 % of the structure, but A can be very different from C because they share only 40 % of structure, and in this case, any clustering of A, B and C will be unsatisfactory: one has to look at the whole dissimilarity of the A, B and C fragments.

None of the approaches seen so far are then completely satisfactory: by presupposing a one-to-one total alignment, we miss the tolerance to indels and by creating a partial alignment between residues we miss the measure of the non aligned part of the structure while introducing arbitrary parameters.

We propose here a novel dissimilarity, named ASD (for Amplitude Spectrum Distance), that overcomes these issues by using the Fourier transform to compare the fragments at a global shape level without explicit structure superimposition. More precisely, ASD measures the *whole* dissimilarity between two fragments as the distance between the amplitude spectra of the discrete Fourier transform of their *C*_*α*_ distance matrix. ASD is computable with a tractable *exact* algorithm (complexity in *O* *N* ^2^ log *N*). Moreover, ASD is a *pseudometric*: it respects the triangle inequality (TI) what provides two main advantages for applications. A computational one, since TI enables to design efficient nearest neighbor retrieval and classification algorithms (see [17]). And a qualitative one, since as pointed out by [18], inter-fragments scores that respect TI provide more meaningful intermediate comparisons and permits a better classification when clustering protein structures (see [19]). Indeed, in order to cluster protein fragments, if a fragment *A* is similar to a fragment *B* (i.e. they are in the same cluster *X*), then for a third protein fragment *C*, say very close to *A*, should also belong to cluster *X*, so that the dissimilarity between *A* and *C* should also be low, what is ensured by TI.

In this paper, we first introduce ASD formal definition and present its properties that makes it suitable for protein fragment comparison. We present then some variants of ASD: a padded version to compare shifted fragments, a normalized version with respect to the length of the fragments and a family of truncated versions enabling to decrease slightly the precision for faster computation. We finally present experiments in which we compare ASD to reference scores: RMSD, RMSD_d_, BC and TM. Let us note that neither DaliLite [20] nor DALIX could have been used for experimental comparison since the first one cannot handle so small fragments and, as shown by preliminary experiments, the second one was too slow for so many pairwise comparisons.

## Methods

We introduce here the formal definition of ASD and present its main properties before introducing some variants of this measure.

### Definition of ASD

We limit ourselves here to backbone structure comparison of two protein fragments. Formally, we identify a protein *P* of *N* residues with a sequence *p*_1_,...,*p*_*N*_ of points in the three-dimensional Euclidean space $\mathbb {R}^ 3$ representing coordinates of the backbone alpha carbons.

We denote by *D*_*P*_ the distance matrix of a protein *P*, whose coefficients are given by:

(1) $$ {D_{P}}_{i,j} := d(p_{i},p_{j})  $$

where *d* is the usual Euclidean distance of $\mathbb {R}^{3}$.

We denote by $\mathcal {F}M$ the two-dimensional unitary discrete Fourier transform [21] of a *N*-square matrix *M*. We recall that:

(2) $$ \mathcal{F}M_{m,n}:=\frac{1}{N}\sum\limits_{p=1}^{N}\sum\limits_{q=1}^{N}M_{p,q}e^{-2i\pi\left(\frac{(p-1)m}{N} + \frac{(q-1)n}{N}\right)}  $$

We denote by |*M*| the matrix whose coefficients are the *moduli* of the coefficients of matrix *M* ; meaning that:

(3) $$ \forall\; 1\leq i,j\leq N,\;|M|_{i,j}:=|M_{i,j}|  $$

We define the following dissimilarity between two protein fragments *P* and *Q* by considering the distance between the *amplitude spectra* of the associated distance matrices:

#### **Definition1** (Amplitude Spectrum Distance).

(4) $$ \text{ASD}(P,Q):= ||\;|\mathcal{F}D_{P}|-|\mathcal{F}D_{Q}|\;||_{2}  $$

where ||.||_2_ is the usual 2-norm:

(5) $$ ||M||_{2}:=\sqrt{\sum\limits_{1\leq i,j\leq N}{} |M_{i,j}|^{2}}  $$

Exact value of ASD can be computed efficiently by $\ensuremath {O\left (N^{2} \log N\right)}$ algorithm [22].

The idea behind this definition is that we do not compare one-to-one *C*_*α*_ distances of proteins, but rather global features (namely the components of the spectra) to assess protein similarity. By focusing on their amplitude and forgetting the phase of the signal, this comparison is more tolerant to insertions/deletions/shifts and enables this way to score more meaningfully intermediates values as shown in the experiments part.

### Properties of ASD

We present here theoretical properties of ASD. All the demonstrations are given in Additional file 1 and Additional file 2.

#### Properties for structural comparison

##### Invariance by isometric transformation

By translating and/or rotating a protein fragment, one would like to keep its similarity with other fragments unchanged. Actually, like any score based on internal distances such as RMSD _*d*_ or DALI’s score, ASD is unchanged by any isometric transformation and thus by fragment translation, rotation or symmetry.

These scores being invariant by mirroring, it may be critical for some applications to distinguish mirrored matches from the classical ones. For any pair of fragments *P* and *Q* assessed to be similar by such a score, this can be done simply by computing the sign of the determinant *d* *e* *t*(*P* ^⊤^ *Q*) where *P* and *Q* are the *N*×3 matrices of the *C*_*α*_ coordinates: a positive determinant shows that it is not a mirror, while a negative one indicates a better superimposition by mirroring one of the two structures [23].

##### Small sensitivity to small changes

ASD can qualified as a gradual dissimilarity since applying small deformations over a protein structure will result at most into a proportional change of ASD.

More formally, a “small” deformation of a fragment can mathematically be captured by a function $f\!\!:\!\!\mathbb {R}^{3}\longrightarrow \mathbb {R}^{3}$ such that $\forall x \in \mathbb {R}^{3}, ||x-f(x)||_{2} \leq \epsilon $. For arbitrary proteins *P*,*Q* and for a such small deformation *f* we can show that the following bounds hold:

(6) $$ \text{ASD}(P,Q) - 2N\epsilon \leq \text{ASD}(f(P),Q) \leq \text{ASD}(P,Q) + 2N\epsilon  $$

##### Euclidean bound and coherence with RMSD_d_

Let *P*,*Q* be two protein fragments that are assumed to be similar without mismatch (meaning that one can align sequentially all the residues one-to-one).Given that RMSD_d_(*P*,*Q*) can be defined as (see [11] for more details):

(7) $$ \text{RMSD}_{\mathrm{d}}(P,Q):=\sqrt{\frac{1}{\binom{N}{2}} \sum\limits_{i < j < n} ({D_{P}}_{i,j} - {D_{Q}}_{i,\,j})^{2}}  $$

We can then bound ASD by RMSD_d_(*P*,*Q*):

(8) $$ \text{ASD}(P,Q) \leq \sqrt{\binom {N} {2}}\;{RMSD}_{\mathrm{d}}(P,Q)  $$

Besides this bound relative to RMSD _*d*_, in the section, experimental support of the nice correlation between classical RMSD and ASD in case of totally aligned (one-to-one) fragments.

#### Specific properties of ASD

##### Invariance by circular permutation

By defining *P*≫*s* to be the protein *P* circularly shifted by *s* residues (such that *D*_(*P*≫*s*)_=*D*_*P*_≫*s*), we get:

(9) $$ \text{ASD}(P,P\gg s) = 0  $$

This property will show its importance when dealing with the padded extension of ASD in the next section.

##### Invariance by sequential inversion

As ASD compares, literally speaking, sequences of points in a 3-dimensional space, no matter the direction of the sequence, if they are superimposable they are considered as similar.

Formally, let us denote by $\overline {P}$ the sequential inversion of a protein *P*=(*p*_1_,…,*p*_*N*_), *i.e.* $\overline {P}=(p_{N},\ldots,p_{1})$. Since the distance matrix $D_{\overline {P}}$ of a sequentially inverted protein *P* is the sequential inversion of matrix *D*_*P*_ such that ${D_{\overline {P}}}_{i,j}={D_{P}}_{N-i+1,N-j+1}$, we can show that:

(10) $$ \text{ASD}(\overline{P},P) = 0  $$

And for arbitrary proteins *P*,*Q*:

(11) $$ \text{ASD}(\overline{P},Q) = \text{ASD}(P,Q)  $$

This property enables to retrieve protein fragments that have the same conformation without taking into account the direction of the sequence. That property, for the best of our knowledge, only appears in non-sequential aligners (such as MICAN [24]) and that are thus very expensive to compute. See in the section for an example of a structural match with one reversed sequence.

##### ASD is a pseudometric

Being a pseudometric can be of great interest for designing efficient algorithms since this property, especially the triangle inequality, is often mandatory for pruning the search space of a nearest neighbor algorithm like in [17].

For three arbitrary proteins *P*,*Q*,*R*, one can show:

- ∀*P*,ASD(*P*,*P*)=0
- ∀*P*,*Q*,ASD(*P*,*Q*)=ASD(*Q*,*P*)
- ∀*P*,*Q*,*R*,
  ASD(*P*,*R*)≤ASD(*P*,*Q*)+ASD(*Q*,*R*)

Thus, ASD is a pseudometric.

Yet, ASD is not a metric over the fragments since we can have *A* *S* *D*(*P*,*Q*)=0 for two *different*proteins *P* and *Q* (taking for example *Q* to be the mirror of *P*, one gets *A* *S* *D*(*P*,*Q*)=0, but *P*≠*Q*).

### ASD variants

#### Padded ASD

To gain flexibility with respect to the fragments alignment, padded matrices can be used to return the best ASD with respect to shifting them.

Formally, we denote by $\widetilde {\text {ASD}}$, the pseudometric obtained by applying ASD on padded matrices:

(12) $$ \widetilde{\text{ASD}}(P,Q):= ||\;|\mathcal{F}\widetilde{D_{P}}|-|\mathcal{F}\widetilde{D_{Q}}|\;||_{2}  $$

where $\widetilde {D_{P}}$ and $\widetilde {D_{Q}}$ are “padded” versions (both of dimension *N*=*N*_*P*_+*N*_*Q*_, padded with zeros) of the matrices *D*_*P*_ and *D*_*Q*_ (of dimensions *N*_*P*_ and *N*_*Q*_ respectively).

**Fig. 1:**
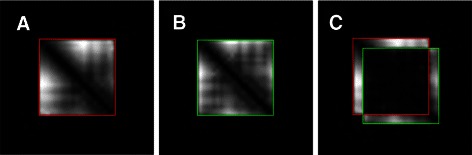
**a** Padded distance matrix for fragment 34:54 of Astral domain d1amk__ ; **b** Padded distance matrix for the same domain but at shifted positions 37:57 **c** Difference of the two matrices in their optimal alignment

We can then establish a theoretical bound of the $\widetilde {\text {ASD}}$ dissimilarity between two protein fragments *P* and *Q* that superimpose exactly over a consequent subpart of them as in example shown in Fig. 1. One can show that:

(13) $$ \widetilde{\text{ASD}}(P,Q)\leq || D_{P\setminus Q}||_{2}  $$

where *D*_*P*∖*Q*_ is the difference of the distance matrices in the *optimal alignment* as illustrated in Fig. 1 c, meaning that, at most, $\widetilde {\text {ASD}}$ measures *only where P and Q differ*.

*Since* $\widetilde {\text {ASD}}$ *is more practical than the original ASD while sharing the same properties, we will use it hereafter and ASD will denote this padded variant in the sequel of this document.*

#### Normalized ASD

As shown in Fig. 6 a, the distribution of ASD values is dependent of the fragments’ length. We introduce here a new normalization of ASD named NASD (for “Normalized ASD”) to overcome this issue.

We define NASD between two protein fragments *P* and *Q* as the following pseudometric:

(14) $$ \text{NASD}(P,Q):=\left|\left|\;\frac{|\mathcal{F}D_{P}|}{||D_{P}||_{2}} -\frac{|\mathcal{F}D_{Q}|}{||D_{Q}||_{2}}\;\right|\right|_{2}  $$

NASD performs well to normalize the scores with respect to the length of the fragments (see Fig. 6 b). This comes at the price of a small information loss caused by the *a priori* distance matrices normalization, as it may be seen in the experiment on zinc finger retrieval presented in the section which does not require length normalization and shows better results for ASD than NASD. We observed moreover on fragments of length 20 that the Pearson correlation coefficient of NASD with ASD was only 0.53, but that they were nevertheless well correlated for small values, with a Pearson correlation coefficient of 0.9 for values of ASD below 1000 (see Fig. 7 a).

#### Truncated ASD

When computing ASD, we use the 2-norm over the module of each Fourier coefficients of the distance matrix. That is to say that computing ASD requires to compare all the Fourier coefficients.

When computational cost matters, it is possible to compare only a small part of them. As Fig. 7 b suggests, we can significantly reduce the computational cost by slightly reducing the precision of ASD. Indeed, Fig. 7 b shows the difference obtained by computing ASD over 40×40=1600 coefficients versus 5×5=25 coefficients. The Pearson correlation coefficient is as high as 0.95.

## Results and discussion

To better understand how ASD compares empirically to classical scores, we have carried out several experiments that we present here.

We first study the distribution and the significance of the scores and observe a good correlation between ASD and RMSD for similar fragments (i.e. that are found totally superimposable by the structural alignment tool Fr-TM-align). We exhibit then explicit examples of divergence between ASD and RMSD leading us to identify 4 causes of disagreement between them. The ability of ASD to retrieve structures with very similar backbones, but in a main-chain reverse order, is illustrated on concrete examples. This property is rare among scores but may be structurally meaningful as pointed out by [25].

We compare then ASD, NASD, RMSD, BC and TM score on a realistic task of related fragment retrieval experiment. We mimic a classical scenario where a first fragment of interest is known and the goal is to find all the other structurally related fragments. The experiment is based on a zinc finger (ZF) family which has been been well studied and annotated and is thus a good basis for comparing safely the different methods: from a given ZF structure, that we call a *seed*, we want to retrieve all the other ZF fragments contained in a dataset mixing true ZF fragments and random fragments from a representative dataset of proteins. The ZF pattern presenting several insertion sites, this is a good test to compare how these scores can deal with insertions or deletions: in this experiment, ASD achieves a significantly better precision for high recalls, showing that it is well-suited for the retrieval of distant related fragments even in the presence of indels.

Finally, we show the relevance of ASD for fragment clustering tasks. First, we consider the set of complementarity-determining regions of immunoglobin (CDR) fragments, that are well known for their very divergent sequences, and show how ASD is able to detect structurally related CDR fragments which target potentially related antigenes. We then give another example of unsupervised classification of domain linkers, and show how the hierarchical clustering using ASD directly catches the manual classification done by [26].

Before presenting the results in details, we introduce first the datasets used.

### Datasets

To perform all-against-all fragment comparisons in reasonable amounts of time for each score on small but representative sets of fragments, we built the **SkF **_***N***_ datasets for *N* equal to 20,30,40,50 and 60 by extracting respectively all (overlapping) fragments of length *N* from the 40 protein domains from the classical "Skolnick data set" described in [27].

For the ZF fragment retrieval experiments, we used the PDB files listed as 3D cross-references in *PS00028* file from Prosite’s Release 20.99 [28] for C2H2 zinc finger motif C-x(2,4)-C-x(3)-[LIVMFYWC]-x(8)-H-x(3,5)-H. Let us remark that C2H2 motif can match regions of different lengths due to the flexible size of the gaps. To enable fixed-length comparison and retrieval of the fragments by the different methods, we extracted all the fragments of 23 residues (ensuring to cover extensively all the ZF sites) starting at the beginning of each pattern match (at the first C). When several models were present in the PDB file, we used only the first model of the structure. By visual inspection we discarded the fragment from residue 18 to 41 in the PDB structure 2MA7 that exhibits a linear shape unlikely to be a ZF. The resulting set of ZF fragments is named **ZF**. To build a representative control set, we extracted 64 Astral protein domains by sampling randomly 16 protein domains in each of the 4 SCOP classes (all alpha, all beta, alpha/beta,alpha+beta) from the Astral 2.03 database [29]. From these domains, we extracted all (overlapping) fragments of 23 residues (the length of the fragments in **ZF**). Finally, we removed PDB files of fragments that have alternative *C*_*α*_ atoms coordinate for one residue position. We denote by **Astral64** the resulting 10,587 protein fragments dataset and we denote by **Astral64+ZF** the dataset consisting in merging **Astral64** and **ZF**.

For the CDR clustering experiment, we used the 559 L1-CDR fragments of the database SAbDab described in [30]. 207 of these 559 fragments had an attributed cluster in the database.

For the domain linker clustering experiment, we used the database described in [26] that contains 1279 fragments, whose length ranges from 2 to 58 residues, 50 % of them having less than 9 residues.

All the datasets used in the experiments can be accessed at http://www.irisa.fr/dyliss/public/ASD/.

### Scores distributions

#### Significance of ASD values

**Fig. 2:**
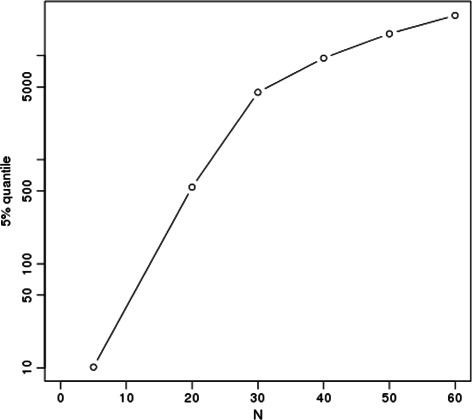
Value of the 5 % quantile of the ASD distribution over **Sk **_***N***_

Like RMSD, the significance of ASD values depends on the fragment length. When comparing all protein fragments of **SkF **_***N***_ using ASD, we ended with the statistical data summarized on Fig. 2. With respect to this empirical evaluation on this dataset, we can see for instance that for a protein fragments of size 23, the 5 % quantile of the distribution of ASD is about 1700, i.e. ASD values smaller than this threshold will correspond to a good similarity.

#### Correlations between scores

We ran over the **SkF**_**20**_ dataset an “all-against-all” comparison using RMSD, TM-score, NASD and ASD, corresponding to a significant set of 15,026,162 fragment comparisons for each score.

**Fig. 3:**
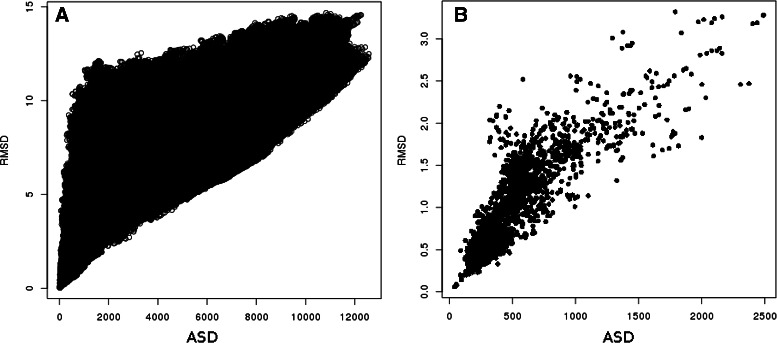
Coherence between ASD and RMSD. **a** Distribution of RMSD versus ASD over **SkF**_**20**_, Pearson correlation coefficient is of 0.65; **b** Correlation between ASD and RMSD for totally aligned fragments of **SkF**_**20**_ by Fr-TM-align. Pearson correlation is of 0.85

On the plot of RMSD vs. ASD on Fig. 3 a one can see that ASD spreads the distribution of the RMSD over intermediate values. However, Fig. 3 b shows that there is a good correlation between RMSD and ASD when considering only the fragments which are similar on their whole length in the **SkF**_**20**_ dataset (i.e. when two fragments are totally aligned by the alignment tool Fr-TM-align).

**Fig. 4:**
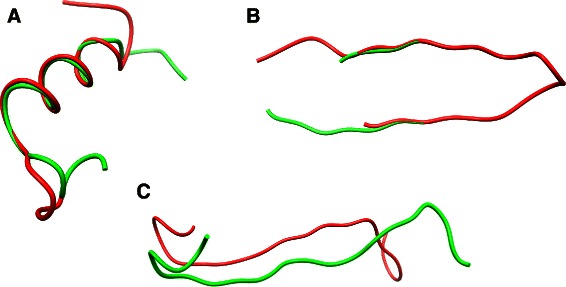
Disagreements with RMSD (similar fragments for ASD but not for RMSD): scores computed on the whole fragments and best manual (sub) alignment illustrated above. **a** Astral d2lvfa, residues 95:117 (red) and Astral d3ruac, residues 48:70 (green), RMSD is 4.8 and ASD is 1446.7; **b** Astral d1x7sa, residues 78:100 (red) and residues 82:104 (green) RMSD is 9.3A and ASD is 1472.3; **c** Astral d3rufa, residues 180:202 (red) and Astral d3w29a, residues 239:261 (green), the RMSD is 4.9 and ASD is 1357.8
- The backbone ordering is reversed, so that the RMSD becomes good when we reverse the ordering of the backbone residues (see “Reverse ordered structure” section below and Fig. 8 for concrete examples),
- The structures are mirrored, it is a consequence of taking internal distances as input data,
- The structures have a very good fit over a subpart which is shifted in sequence, and thus, RMSD makes a meaningless comparison. See Fig. 4 a for a concrete example of common subpart superimposition in two different proteins. Figure 4 b shows out an example of *sequence shift* impact for the *same* hairpin when comparing fragments corresponding to a shift by 4 residues of the sequence window.

**Fig. 5:**
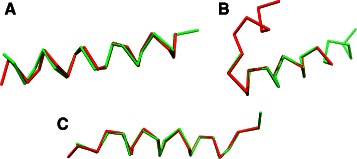
Disagreements with TM-score: scores computed on the whole fragments and best manual (sub)alignment shown above. **a** Astral identifiers d1b71A_ residues 124:144 (in red) and d1psA_ residues 113:133 (in green) are similar for ASD (562) but not for TM-score (0.44). **b** Astral identifiers d1fha__ residues 109:129 (in red) and d1rcd_ residues 102:122 (in green) are similar for TM-score (0.60) but not for ASD (2995), in manual alignment, 14 residues over 20 are aligned. **c** Astral identifier d1amk_, residues 136:156, in red, and d1tri_ residues 126:146 are examples of similar fragments correctly scored by both ASD and TM-score that the heuristic of Fr-TM-align fails to align: TM-score on whole fragments is 0.7, while Fr-TM-align returns a TM-score of 0.27 by aligning only 11 residues

By analyzing the outliers of the distribution ASD vs. RMSD, we ended with different causes when ASD gives low dissimilarity while RMSD gives a high one:

- The two structures have a rough similar global shape but present local variations, preventing them from being superimposed (see Fig. 4 c for an example),

The outliers of the distribution ASD versus TM-score were harder to classify. The extreme examples are couples of structures that are main chain reversed, so that the TM-score is bad while the ASD gives it a low distance. The other main difference lies in the intermediate scoring, where TM-score seems to focus on local similarities, while ASD seems to score more the global shape similarity. For example on Fig. 5, one can see that ASD assess for much more similarity (and inversely for TM-score) in two helices that are globally the same but whose atoms present local variations (Fig. 5 a) than to the two structures that are locally very similar over a subpart but globally divergent (Fig. 5 b). Other examples for the ZF fragment retrieval experimentation are available in Fig. 7.

**Fig. 6:**
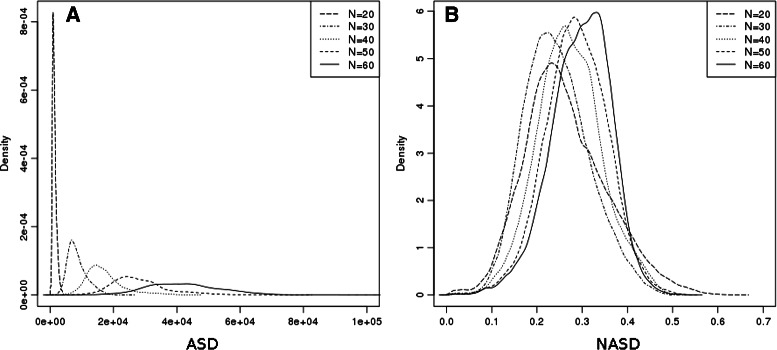
Empirical probability density (over the **Sk**_*N*_ dataset) of ASD (**a**) and NASD (**b**) for fragments of respective length *N*=20, 30, 40, 50 and 60 residues from **SkF **_***N***_

**Fig. 7:**
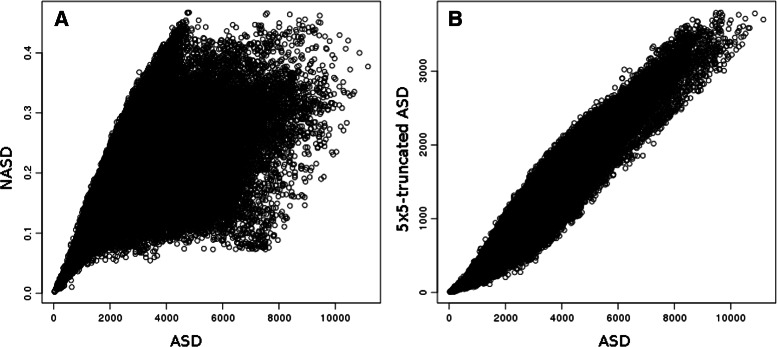
Correlation between ASD and its variants over all fragment pairs of **SkF**_**20**_ **a** ASD vs NASD, Pearson correlation coefficient is 0.53. but reaches 0.9 when zooming on values of ASD below 1000 **b** ASD computed using all the 40×40 Fourier coefficients vs truncated version of ASD computed using only 5×5 first Fourier coefficients, Pearson correlation is 0.95

**Fig. 8:**
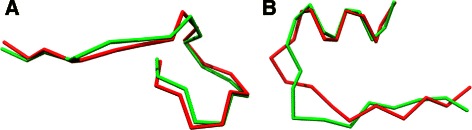
Examples of “head to tail” structural alignments found by ASD in SkF_20_. **a** Fragments (Astral d1b9bA, residues 60:80, in red, and d1treA residues 60:80 in green), with very good ASD value (358) and bad TM-score (0.38), which can almost be superimposed when one sequence is in reverse order of the other. **b** Fragments (Astral d1qmpD, residues 74:94, in green, and d1qmpC residues 63:83, in red) with a fair ASD value (797) but poorly scored by the TM-score (0.11) that have a rough similar global shape with local variation and main chain reversal

**Fig. 9:**
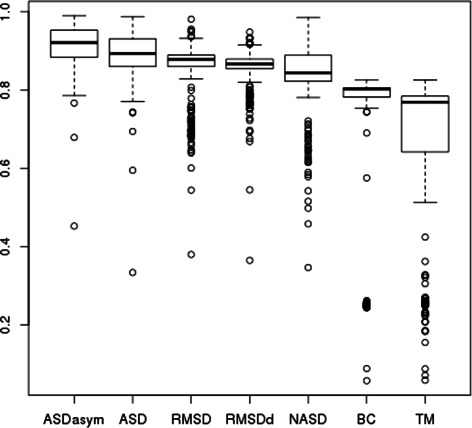
Box plot of PR AUC for all ZF seeds

**Fig. 10:**
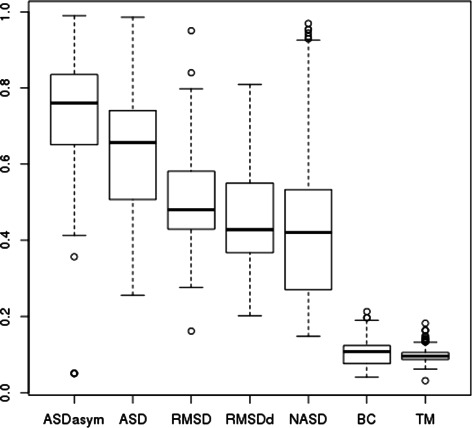
Box plot of precision for 90 % recall for all ZF seeds

**Fig. 11:**
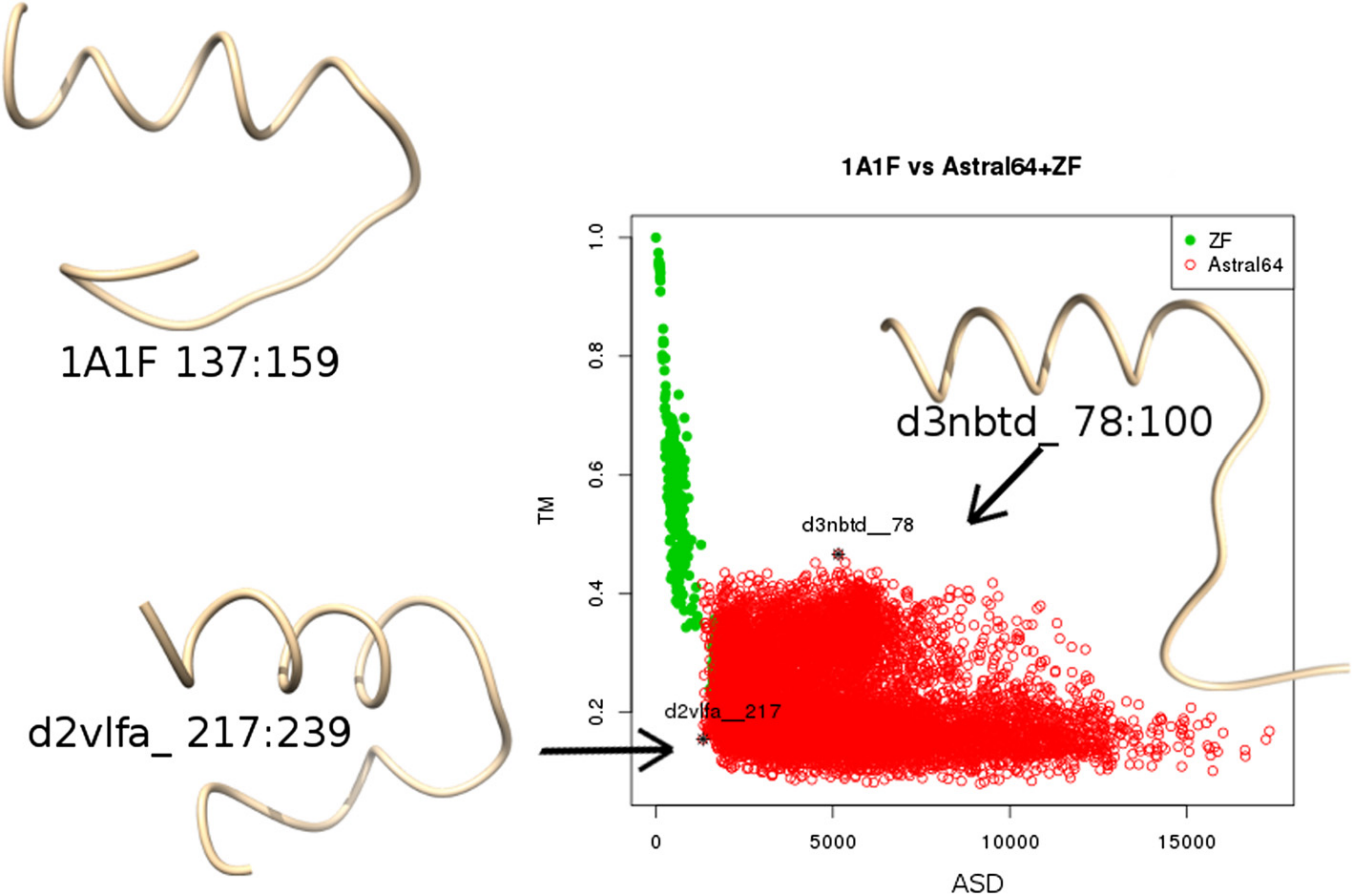
ASD vs TM scores for the comparison of an arbitrary ZF fragment seed (PDB 1A1F, residues 137:159) with Astral64 (red dots) or ZF (green dots) fragments. Structure of the seed is shown as well as the structures of Astral d3nbtd residues 78:100, the first false positive by TM-score among the true negatives of ASD, and Astral d2vlfa residues 217:239 the first false positive for ASD among the true negatives of TM-score

Finally, as already discussed before, we can also see on this dataset the good normalization of NASD with respect to length compared to ASD (Fig. 6) even if some information is lost (7 a) and the good correlation of ASD with its truncated variant in Fig. 7 b, so that the computing time can be drastically reduced when speed is more important than high accuracy.

### Reverse ordered structure

As shown in [25], structural similarity of convergent enzymes may occur in a “non-sequential way”. Indeed, one can almost superimpose two structures but the secondary structure will not be sequentially linked in the same way and may be main-chain reversed in the sequence. Thus, comparing two structures up to the sequential direction may be useful in this kind of enzymes.

ASD is able to mine protein fragments regardless to the sequential order. To illustrate this property, Fig. 8 a presents some concrete instances of fragments from **SkF**_**20**_ found similar by ASD that superimpose well when aligning residues in reverse order of each other.

### Zinc finger retrieval

We present here how the different structural scores and measures compare on a realistic task of ZF fragment retrieval: using an arbitrary ZF fragment as a seed, we ran a nearest neighbor retrieval experiment over **Astral64+ZF**, considering the fragments of **Astral64** as *false* hits and the fragments of the **ZF** dataset as *true* hits. There are 10,587 fragments in **Astral64** (which are considered as the false hits), and 321 fragments in the **ZF** dataset (which are considered as true hits).

**Fig. 12:**
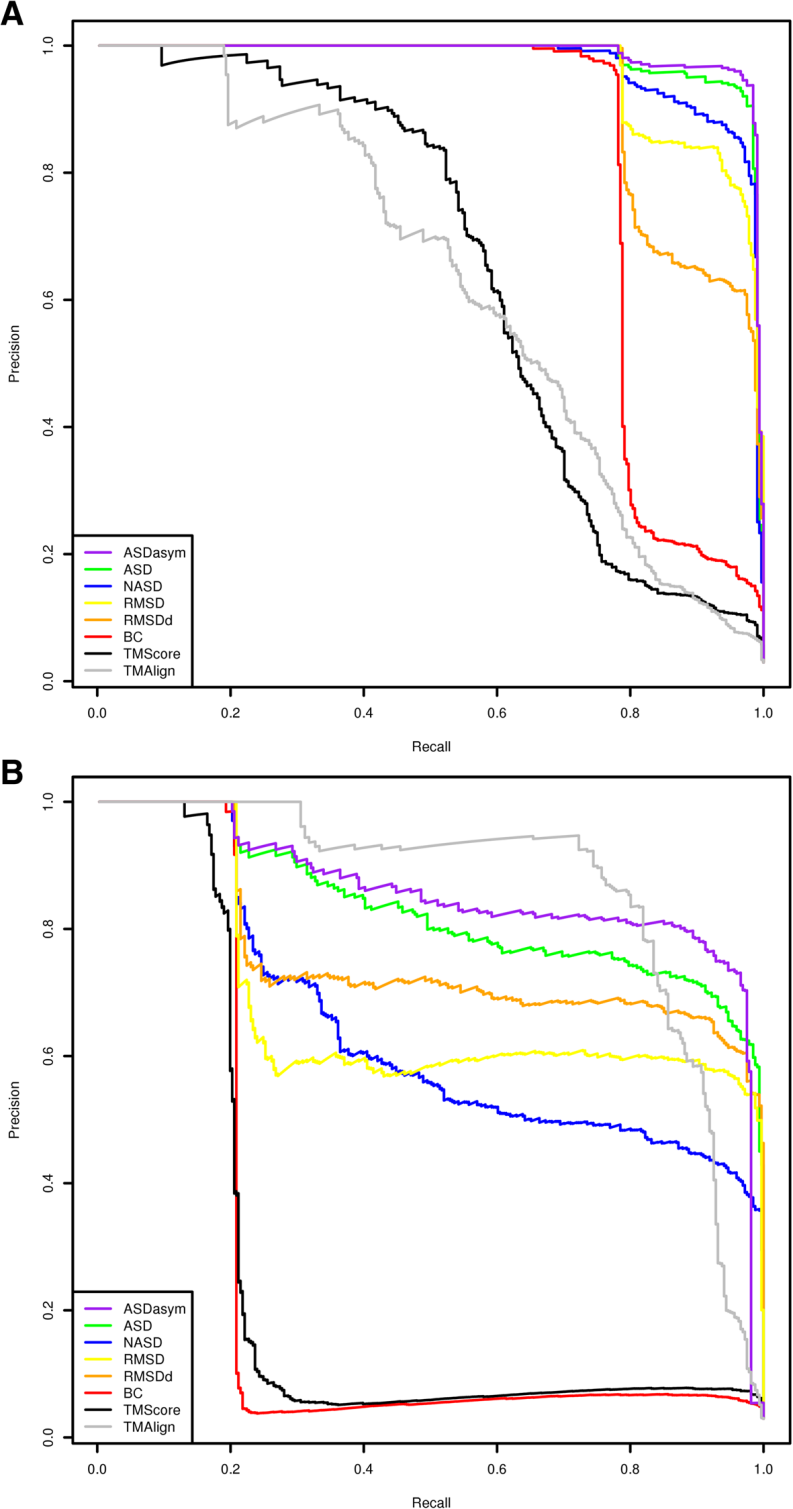
Examples of Precision-Recall curves of each score for ZF fragment retrieval over Astral64+ZF: ASD, NASD, ASDasym, RMSD, RMSDd, BC and TM scores are computed on whole fragments while TMAlign’s curve is based on the TM-score of the subpart of the fragments that is aligned by Fr-TM-align. **a** using 1BBO residues 32:54 as seed and **b** using 1A1G residues 107:130 as seed

We made the experiment in a jack-knife way: every ZF fragment is used iteratively as the seed for retrieving ZF fragments among the whole **Astral64+ZF** dataset enabling to plot the corresponding precision-recall curve. Two typical examples of the precision-recall curves obtained during the experimentation (respectively for seed 1BBO, residues 32 to 54, and 1A1G, residues 107 to 130) are shown in Fig. 12.

Computing the area under each precision-recall curve (PR AUC) [31] enables to compare the performance of the scores, the bigger being the better and the optimal value of PR AUC being 1.0 (perfect precision for perfect recall). Fig. 9, shows the average PR AUC on all the seeds for each score. We see that ASD has a better PR AUC than any other of the tested scores. At the second place, RMSD and RMSDd perform quite well in these experiments. The improvement brought by ASD with respect to RMSD is significant (the Welch *t*-test between ASD and RMSD values has a *p*-value of 1.5.10^−10^) and is mainly explained by the good precision obtained for high recalls as shown in Fig. 10 (ASD has a mean precision 26 % higher than the RMSD for 90 % of recall). This excellent recall contrasts with BC score which is very specific and retrieves only close fragments without indels, showing a complementary ability to discriminate better at finer scales.

TM-score is ranked last for this fragment retrieval task (see Fig. 11 for an example providing a more detailed view on its distribution with respect to ASD and respective instances of false positive hits). We tested also Fr-TM-align to see how a tool searching for (sub)alignment compares with the other scores. Fr-TM-align was too slow to perform the complete experimentation but we were able to run it on a few seeds (two precision recall curves are shown in Fig. 12) and we observed very irregular performances, ranging from worse (PR AUC of 0.62 for 1BBO residues 32:54 as ZF seed while of 0.98 for ASD and 0.83 for BC) to better (PR AUC of 0.86 for 1A1F residues 107:130 as ZF seed while of 0.83 for ASD and 0.85 for ASDasym, introduced below, but dropping rapidly for 90 % of recall to a precision of 0.58 compared to 0.72 and 0.78 for ASD and ASDasym respectively).

Finally, since ASD is invariant by mirroring, we ran a complementary experiment extending ASD with an additional test to discard anti-symmetric false positives. To this end, we used the determinant introduced in the section describing ASD properties. We ranked first according to ASD the instances that have a positive determinant with the seed, and behind (once again according to ASD), the instances whose determinant was negative. The results of this ranking are labeled "ASDasym" on the Figs. 9 and 10. We see an additional improvement with respect to ASD: ASDasym has a mean precision 44 % higher than the RMSD for 90 % of recall.

### Classification of CDR L1

Antibodies are proteins that play a key role in immunitarian system by binding a specific antigen. Actually, only a small part of their three-dimensional structure, called complementarity-determining regions of antibodies (CDR), determines the antigene they bind. Each CDR is composed of six protein fragments named L1,L2,L3– fragments on the light chain of the antibody – and H1,H2,H3– fragments on the heavy chain of the antibody. The paper [30] presents the SAbDab database of antibodies that includes the most recent classification of CDR established by [32].

We present here, as an example, an automatic clustering of L1 CDR fragments relying on ASD score and we compare it to the results of [32]. Since these fragments are of different lengths, usual scores are not able to perform the structural comparison of the different fragments. To give a way of comparison we compared our results to what can be obtained with the structural aligner TM-align [14].

**Fig. 13:**
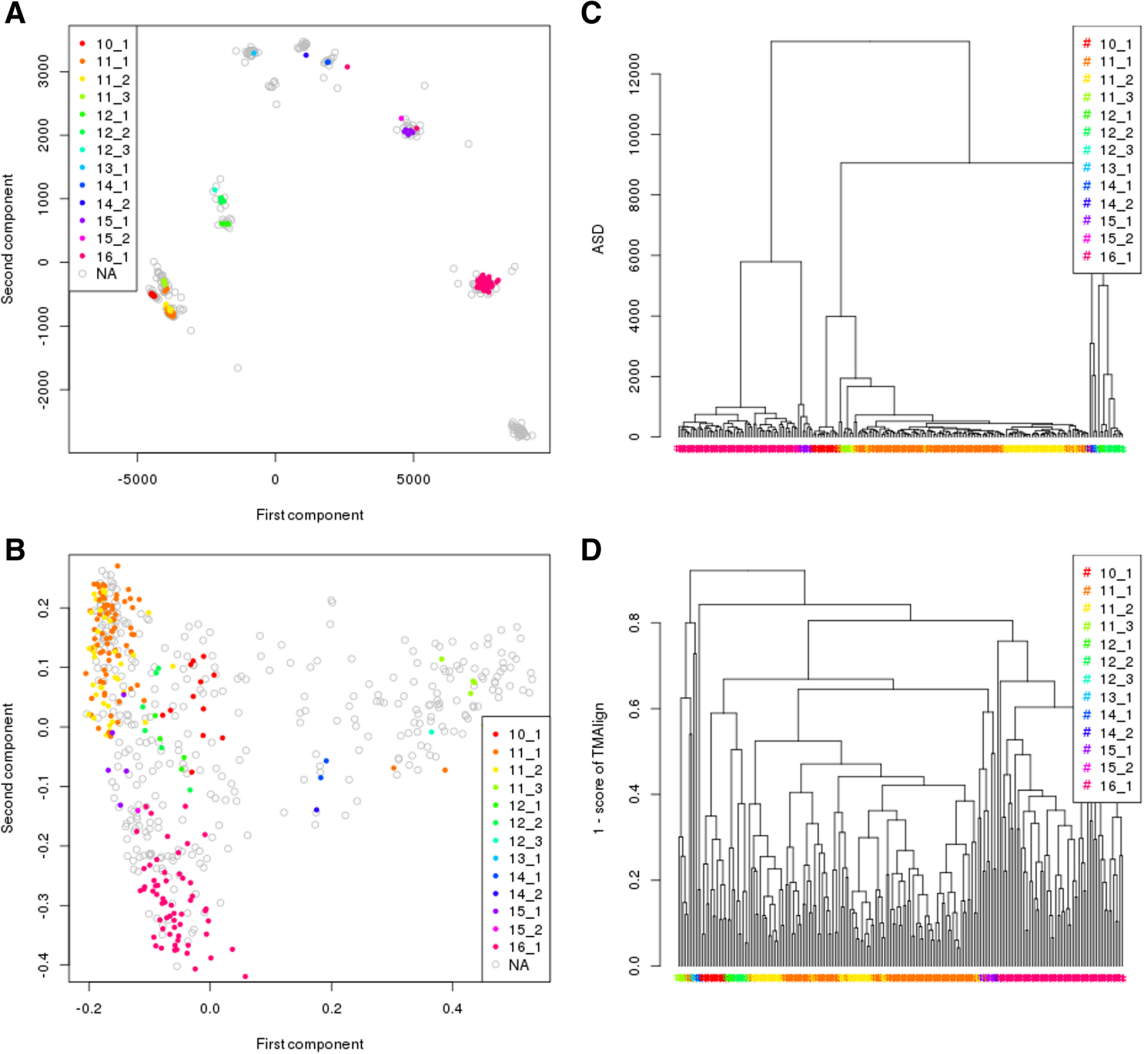
Two-dimensional representation of similarities between L1 CDR as computed by ASD (**a**) and by TM-align (**b**). Hierarchical classification of L1 CDR using ASD (**c**) and TM-align (**d**)

Figure 13 a and b present the multi-dimensional scaling (MDS) of the dissimilarity between the fragments as computed by ASD and TM-align respectively. MDS is a practical way of visualizing high-dimensional data into lower dimensions (here of dimension 2): it gives the best planar representation such that the closer are two points in the plane, the more similar the fragments are considered. The colors of the L1-CDR fragments refer to the definitions in the paper [32]. The NA grey circles are L1-CDR fragments with no cluster attribution in the SAbDab database [30]. We can see, especially in contrast to TM-Align, that ASD makes very sharp clusters that agree mostly with the classification of [32], and that unannotated L1-CDR in grey can easily be assigned to existing clusters or are candidate for a new cluster (see cluster at bottom right corner of the plot).

Figure 13 c and d show a standard complete-linkage hierarchical classification using ASD and TM-align respectively. We can see that the clusters are more scattered with TM-Align than with ASD. This means that clusters are more robust and that the association of a new structure to a cluster is easier using ASD.

Davies-Bouldin index [33] measures the quality of a clustering and can be used to cut the dendrogram of a hierarchical clustering by looking at its local minimum value. We found local minimum values of 0.6 and 0.2 for TM-Align and ASD respectively. As the DB-Index is lower for ASD, the clustering quality is better using ASD. The corresponding cuts lead to 7 clusters in the case of TM-Align with 71.3 % of agreement with the reference classification, while using ASD, it results in 10 clusters having an agreement of 84.0 % with the reference.

Last, the computation time was about 10 times faster with ASD than with TM-align, and could have been sped up furthermore using triangle inequality property.

### Classification of domain linkers

**Fig. 14:**
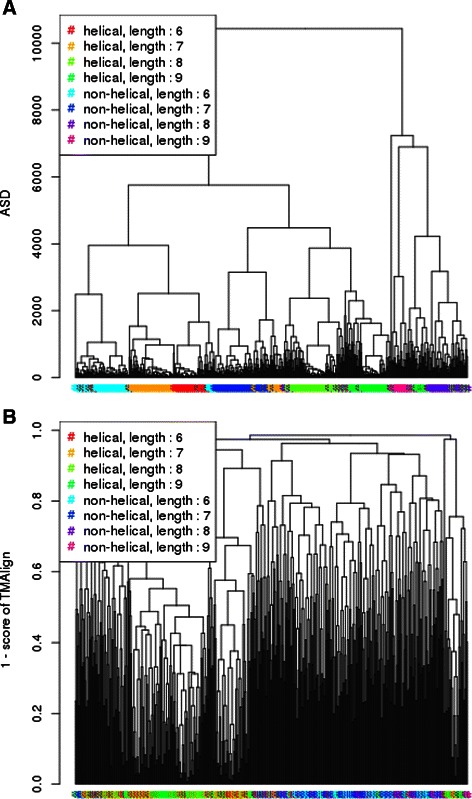
Hierarchical classification of linkers of 6 to 10 residues using ASD (**a**) or TM-align (**b**)

A linker is a structural fragment of protein structure that connects two protein domains together. Linkers are of interest for example in protein engineering when expressing a unique polyprotein that will have several enzymatic function: the different domains should be linked with linkers with specific characteristics (length, flexibility for instance). George and Heringa [26] presents an expert-based classification of linkers found in natural proteins where size and *helical* vs. *non-helical* were the two criteria used for the classification. As in the previous experiment, the fragments are of different lengths, so ASD will be compared once again to TM-align. As TM-align is not able to compare fragment smaller than 6 residues, we show on Fig. 14 the classification of linkers with a length ranging from 6 to 9 residues. We also chose to limit up to 9 residues because the number of available linker structures were too small compared to size of the conformation space, preventing from recognizing any sharp class.

**Fig. 15:**
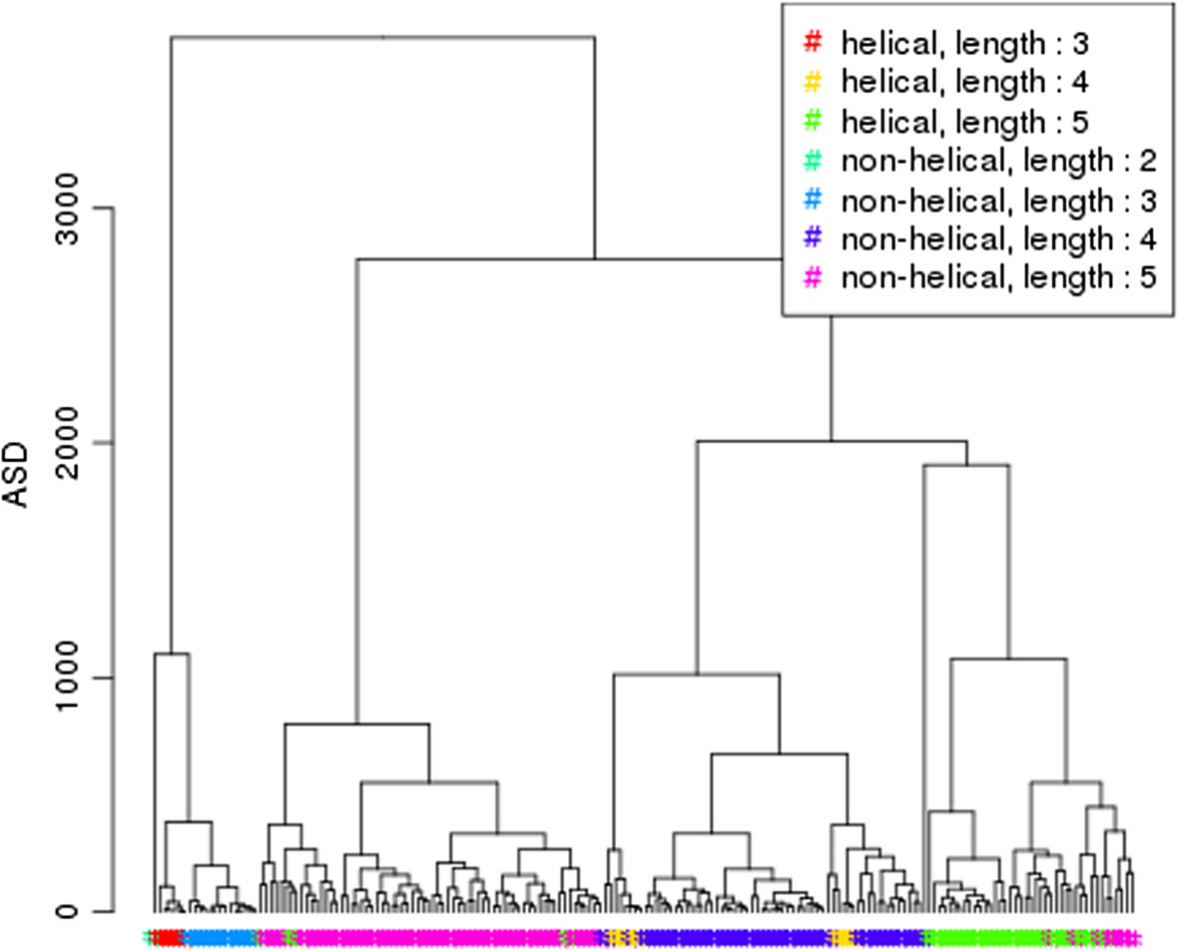
Hierarchical classification of linkers of less than 6 residues by ASD

As a matter of fact, ASD finds the same distinction according to both length and helical/non-helical classes than proposed in [26] while the scores given by TM-align does not show any sharp order. We provide also the classification as done by ASD of linkers smaller than 6 residues on Fig. 15.

## Conclusion

Taking advantage of the power of discrete Fourier transform, we have introduced ASD, an efficiently computable pseudometric measuring the global shape dissimilarity of protein fragments. By comparing the amplitude spectra of the internal distance matrices, ASD performs a more comprehensive comparison than by one-to-one distances between the residues, that makes it tolerant to indels while 1) requiring neither to search for best (sub)alignment nor to introduce ad-hoc parameters (avoiding thus the consequent empirical tuning of quality/length trade-off) and 2) preserving the triangle inequalityproperty.

Several experiments have been presented to assess the relevance of ASD on real fragment comparison tasks. First, through a large set of fragments comparisons, we have seen that ASD is well correlated with classical scores for easy alignment cases and that main disagreements are due either to its flexible comparison of fragments (e.g. tolerant indel and shifts, providing ASD an advantage over the other scores) or to the invariance of ASD by mirroring and reversal (that can be easily bypassed if needed, as in the ZF experiment with the ASDasym ariant).

Second, we have estimated the benefit of ASD with respect to other scores for more difficult cases involving the comparison of distantly related fragments. In the lack of a Gold standard, we have set up an indirect experimental assessment to evaluate the scores on a realistic task: from one instance of a structure of a zinc finger (ZF) fragment, we evaluate how well ASD retrieves all the fragments – including those that carry indels– belonging to the same structural ZF family among non-ZF fragments. This experiment has witnessed a good tolerance of ASD to indels compared to BC score, TMScore and RMSD, and illustrated its usefulness for retrieval applications requiring a high recall on distantly related fragments.

And then, the benefits of ASD when dealing with classification tasks were illustrated by the CDR and domain linker clustering experiments. In both cases ASD is the only score, to the best of our knowledge, which is capable of comparing fragments of different lengths without relying on structural alignment. On these experiments, ASD performs better and faster than the common TM-align aligner and mostly agree with existing classifications built by experts. Moreover, thanks to the sharpness of the clusters derived from ASD, one gets an accurate insight for attributing a cluster to non-classified fragments.

The definition and properties of ASD coupled to these first experiments make ASD a good candidate to fill the current gap in measuring the structural divergence of fragments.

To go further in the study of ASD, carrying out additional practical experiments would help to appreciate the impact and interest of ASD invariance with respect to mirroring and main chain reversal. It would also be interesting to investigate the relevance of ASD for the comparison of whole protein domains.Concerning the possible developments of ASD, the computation time could be sped up for massive comparisons by considering less Fourier coefficients as proposed in the ASD variants section and eventually by weighting them adequately ; but this would require a careful study of the speed gain versus the precision loss.Finally, from a general perspective, we have shown here that the spectra of the distance matrix of a protein fragment contains information for the comparison of fragments. One further direction of research would be to use this information to determine the key elements of the spectra that make some related fragments similar.

An application would be for instance to determine the characteristic spectra of a family of protein fragments to build a dedicated dissimilarity measure enabling a finer retrieval of new members.

## Additional files

Additional file 1
**Detailed-properties-and-proofs-of-ASD.** In this document, we present more formally, with the proofs, the different properties of ASD presented in the current paper. (2003KB PDF)

Additional file 2
**Dataset-ID.** Identifiers of the proteins used in the experiments. (67.4KB PDF)
