# Supplementary material for: Amplitude spectrum distance: measuring the global shape divergence of protein fragments
Source: BMC Bioinformatics. 2015 Aug 14;16:256. doi: 10.1186/s12859-015-0693-y (PMC4535829; doi:10.1186/s12859-015-0693-y)
Supplement: Additional file 1 — Detailed-properties-and-proofs-of-ASD. In this document, we present more formally, with the proofs, the different properties of ASD presented in the current paper. (2003KB PDF) [file 12859_2015_693_MOESM1_ESM.pdf]

Details of the datasets used in the paper "Amplitude Spectrum Distance: measuring the global shape divergence of protein fragments"

C. Galiez, F. Coste

August 4, 2015

In this document, we give the list of the elements used in the **ZF**, **Astral64** and **SkF** datasets used in the paper "Amplitude Spectrum Distance: measuring the global shape divergence of protein fragments". The listing provided in this document can be downloaded at: http://www.irisa.fr/dyliss/public/ASD/.

1 ZF

For the ZF fragment retrieval experiments, we used the PDB files listed as 3D cross-references in *PS00028* file from Prosite's Release 20.99 [1] for C2H2 zinc finger motif C-x(2,4)-C-x(3)-[LIVMFYWC]-x(8)-H-x(3,5)-H. Let us remark that C2H2 motif can match regions of different lengths due to the flexible size of the gaps. To enable fixed-length comparison and retrieval of the fragments by the different methods, we extracted all the fragments of 23 residues (ensuring to cover extensively all the ZF sites) starting at the beginning of each pattern match (at the first C). When several models were present in the PDB file, we used only the first model of the structure. By visual inspection we discarded the fragment from residue 18 to 41 in the PDB structure 2MA7 that exhibits a linear shape unlikely to be a ZF. The resulting set of ZF fragments is named **ZF**, and the PDB identifiers together with the starting residue are given in the table below:

PDB	Res.	PDB	Res.	PDB	Res.	PDB	Res.	PDB	Res.	PDB	Res.	PDB	Res.
1A1F	137	1UBD	327	2EM3	15	2EPR	357	2YT9	392	1ZAA	37	2I13	108
1A1H	107	1UBD	355	2EM5	15	2EPS	415	2YT9	422	1ZAA	7	2I13	24
1A1H	137	1UBD	385	2EM6	15	2EPT	79	2YTA	141	2CSH	40	2I13	80
1A1H	165	1UN6	107	2EM8	15	2EPU	107	2YTB	198	2DLK	41	2JP9	69
1A1I	107	1UN6	137	2EM9	15	2EPV	810	2YTD	15	2DLQ	97	2JP9	9
1A1I	137	1VA3	599	2EMB	15	2EPW	920	2YTF	15	2DMI	22	2JP9	97
1A1J	107	1WIR	18	2EMC	15	2EPY	530	2YTG	15	2DRP	113	2JPA	69
1A1K	107	1X5W	12	2EME	15	2EPZ	507	2YTH	15	2EBT	405	2JPA	9
1A1K	137	1X6E	17	2EMF	15	2EQ0	459	2YTJ	15	2EE8	48	2JPA	97
1A1K	165	1X6E	45	2EMG	15	2EQ1	487	2YTK	15	2EL6	15	2LCE	48
1A1L	107	1X6F	28	2EMK	15	2EQ3	711	2YTO	15	2ELO	12	2LT7	496
1A1L	137	1X6H	18	2EML	15	2EQ4	458	2YTP	15	2ELS	12	2LT7	524
1AAY	137	1XF7	5	2EMP	15	2EQW	414	2YTQ	15	2ELY	15	2LT7	552
1AAY	165	1YUJ	36	2EMW	13	2GLI	106	2YTR	15	2ELZ	15	2LV2	31
1ARD	106	1ZAA	65	2EMX	13	2GLI	202	2YTT	15	2EM0	15	2LVT	32
1ARF	106	1ZFD	44	2EMY	15	2HGH	137	2YU5	15	2EM1	13	2M0E	34
1BBO	32	1ZNF	3	2EN1	15	2I13	52	2YU8	15	2EM2	15	2MDG	6
1BHI	9	1ZR9	45	2EN2	14	2J7J	34	4F2J	473	2EM4	15	2PRT	355
1EJ6	183	2ADR	106	2EN4	15	2J7J	4	4F6M	524	2EM7	15	2PRT	385
1G2D	107	2ADR	134	2EN6	15	2JP9	39	4F6M	552	2EMA	15	2PRT	413
1G2D	137	2COT	21	2EN7	15	2JPA	39	7ZNF	5	2EMH	15	2RPC	123
1G2D	165	2COT	49	2EN8	15	2KMK	32	1A1G	107	2EMI	15	2RPC	93
1G2D	207	2CSE	183	2EN9	15	2KMK	4	1A1G	137	2EMJ	15	2RSH	12
1G2D	237	2CSE	51	2ENC	15	2KMK	60	1A1I	165	2EMM	15	2RSJ	67
1G2F	107	2CT1	18	2ENE	15	2KVF	6	1A1J	137	2EMV	15	2WBS	432
1G2F	207	2CT1	48	2ENF	15	2L1O	8	1A1L	165	2EMZ	15	2WBT	103
1G2F	237	2CTD	65	2ENH	15	2LCE	20	1AAY	107	2EN0	13	2WBT	77
1JK1	107	2D9H	10	2EOE	15	2LV2	59	1ARE	106	2EN3	15	2YRJ	15
1JK1	137	2D9H	41	2EOF	15	2LVR	6	1G2D	265	2ENA	15	2YSP	15
1JK2	137	2DLK	10	2EOG	13	2M0D	6	1G2F	137	2ENT	353	2YT9	364
1JK2	165	2DLQ	10	2EOI	13	2M0F	62	1G2F	165	2EOH	15	2YTE	13
1LLM	106	2DLQ	69	2EOJ	15	2M9A	16	1G2F	265	2EOM	15	2YTI	15
1LLM	206	2DMD	11	2EOK	13	2M9A	44	1JK1	165	2EOS	14	2YTM	15
1P47	107	2DMI	83	2EOL	13	2M9A	74	1JN7	11	2EOV	15	2YTN	15
1P47	137	2DRP	143	2EON	15	2MA7	46	1NCS	34	2EOW	15	2YTS	15
1PAA	134	2EBT	375	2EOO	15	2PRT	325	1NJQ	8	2EOZ	15	3AX1	500
1SP1	5	2EBT	435	2EOP	15	2RSI	39	1P7A	14	2EP0	15	3MJH	43
1SP2	5	2EE8	20	2EOQ	15	2RSI	67	1TF3	15	2EP3	15	3UK3	473
1SRK	10	2EE8	76	2EOR	15	2RSJ	12	1TF6	107	2EPP	294	3ZNF	5
1TF3	45	2EL4	15	2EOU	15	2WBU	402	1TF6	137	2EPQ	385	4F6M	496
1TF3	75	2EL5	13	2EOX	15	2WBU	432	1TF6	45	2EPX	478	4F6N	496
1TF6	15	2ELR	12	2EOY	15	2YRH	13	1VA1	539	2EQ2	683	4F6N	524
1TF6	75	2ELU	12	2EP1	15	2YRK	16	1VA2	569	2GLI	172	4F6N	552
1U85	10	2ELV	12	2EP2	15	2YRM	13	1WJP	72	2GLI	233	4IS1	473
1U86	10	2ELW	12	2EPA	20	2YSO	15	1X3C	30	2GQJ	57	4ZNF	5
1UBD	298	2ELX	10	2EPA	50	2YSV	760	1YUI	36	2HGH	107		

2 Astral64

To build a representative control set, we extracted 64 Astral protein domains by sampling randomly 16 protein domains in each of the 4 SCOP classes (all alpha, all beta, alpha/beta,alpha+beta) from the Astral 2.03 database [2]. The 64 Astral identifiers are listed below:

d1bgab_

d1bz1a_

d1cpcl_

d1ehyb_

d1f7ca_

d1fhqa_

d1gbda_

d1gqcb_

d1i4td_

d1lt6f_

d1s3ca_
d1uppj_
d1urpa_
d1x7sa_
d1x8mf_
d1xtvb_
d1y4vc_
d1y59t_
d2a3wn_
d2ahcb_
d2bkck_
d2c1dd_
d2c7la_
d2hbdb_
d2j73a_
d2o64a_
d2qdsa_
d2uzla_
d2vlfa_
d2xjoa_
d3az9n_
d3b2ja_
d3dcgb_
d3diea_
d3e29d_
d3eqba_
d3euya_
d3fckb_
d3hf9l_
d3hnid_
d3i5vd_
d3jxza_
d3kwaa_
d3l2yg_
d3lele_
d3m1ob_
d3m64a_
d3n6ab_
d3nbtd_
d3nhha_
d3oced_
d3qiha_
d3rdhb_
d3ruac_
d3rufa_
d3uh7b_

d3ux7c_
d3w29a_
d3zxeb_
d4actb_
d4bcqc_
d4ejja_
d4epva_
d4i83f_

From these domains, we extracted all (overlapping) fragments of 23 residues (the length of the fragments in **ZF**). Finally, we removed PDB files of fragments that have alternative C_α atoms coordinate for one residue position. We denote by **Astral64** the resulting 10,587 protein fragments dataset.

3 SkF

The SkF_N datasets for N equal to $20, 30, 40, 50$ and 60 is generated by extracting respectively all (overlapping) fragments of length N from the 40 protein domains from the classical "Skolnick data set" described in [3], and whose Astral identifiers are listed below:

d1amk_
d1aw2A
d1b00A
d1b71A
d1b9bA
d1bawA
d1bcfA
d1btmA
d1byoA
d1byoB
d1dbwA
d1dpsA
d1fha_
d1htiA
d1ier_
d1kdi_
d1nat_
d1nin_
d1ntr_
d1pla_
d1qmpA
d1qmpB
d1qmpC
d1qmpD
d1rcd_
d1rn1A
d1rn1B
d1rn1C

d1tmhA
d1treA
d1tri_
d1ydvA
d2b3iA
d2pcy_
d2plt_
d3chy_
d3ypiA
d4tmyA
d4tmyB
d8timA

4 CDR-L1 and Domain linkers

Identifiers are too numerous to be reported here, see the listings at http://www.irisa.fr/dyliss/public/ASD/.

Author details

References

1. Sigrist CJA, Castro ED, Cerutti, L., Cuche BA, Hulo N, Bridge A, Bougueleret L, Xenarios, I.: New and continuing developments at prosite. Nucleic Acids Research. 2013; 41(Database-Issue): 344–347
2. Chandonia, J.-M.M., Hon G, Walker, N.S., Lo Conte L, Koehl P, Levitt M, Brenner SE. The ASTRAL Compendium in 2004. Nucleic Acids Research 32(Database issue): 189–192 (2004)
3. Lancia G, Carr R, Walenz, B., Istrail S. 101 optimal pdb structure alignments: A branch-and-cut algorithm for the maximum contact map overlap problem. Proceedings of the Fifth Annual International Conference on Computational Biology, 193–202 (2001)
