# Supplementary material for: Amplitude spectrum distance: measuring the global shape divergence of protein fragments
Source: BMC Bioinformatics. 2015 Aug 14;16:256. doi: 10.1186/s12859-015-0693-y (PMC4535829; doi:10.1186/s12859-015-0693-y)
Supplement: Additional file 2 — Dataset-ID. Identifiers of the proteins used in the experiments. (67.4KB PDF) [file 12859_2015_693_MOESM2_ESM.pdf]

Detailed properties and proofs of paper "Amplitude Spectrum Distance: measuring the global shape divergence of protein fragments"

C. Galiez, F. Coste

February 25, 2015

In this document, we present more formally, with the proofs, the different properties of ASD presented in the paper "Amplitude Spectrum Distance: measuring the global shape divergence of protein fragments".

1 Definition and notations

We first recall the definitions and notations used in the paper with a slight change on the indices ranging hereafter from 0 to $N-1$ for practical and readability reasons.

Let P be a protein whose 3D coordinates are $p_0, ..., p_{N-1}$.

The internal distance matrix D_P is defined as:

$$D_{P\,i,j} := d(p_i, p_j) \tag{1}$$

where d is the usual Euclidean distance of $\mathbb{R}^3$.

The two-dimensional unitary discrete Fourier transform ([1]) $\mathcal{F}M$ of a N-square matrix M is defined as:

$$\mathcal{F}M_{m,n} := \frac{1}{N}\sum_{p=0}^{N-1}\sum_{q=0}^{N-1} M_{p,q} e^{-2i\pi(\frac{pm}{N}+\frac{qn}{N})} \tag{2}$$

We denote by $|M|$ the matrix whose coefficients are the *modules* of the coefficients of matrix M ; meaning that:

$$\forall\, 0 \leq i,j \leq N-1,\ |M|_{i,j} := |M_{i,j}| \tag{3}$$

Definition 1 (Amplitude Spectrum Distance) *For two protein fragments P and Q, we define:*

$$ASD(P,Q) := ||\ |\mathcal{F}D_P| - |\mathcal{F}D_Q|\ ||_2 \tag{4}$$

where $||.||_2$ is the usual 2-norm:

$$||M||_2 := \sqrt{\sum_{0\leq i,j\leq N-1} |M_{i,j}|^2} \tag{5}$$

We recall the Parseval theorem that establishes the isometry of the unitary Fourier transform:

$$||\mathcal{F}M||_2 = ||M||_2 \tag{6}$$

For simplicity's sake, we will sometimes present proofs using the uni-dimensional unitary Fourier transform, defined as:

$$\mathcal{F}\vec{x}_n := \frac{1}{\sqrt{N}} \sum_{p=0}^{N-1} \vec{x}_p e^{-2i\pi \frac{pn}{N}} \tag{7}$$

1.1 Properties of ASD

1.2 Invariance by isometric transformation

Like any score based on internal distances, ASD is unchanged by any isometric transformation and thus by fragment translation or rotation.

Formally, if a transformation T over $\mathbb{R}^3$ is isometric, the distance matrix D_{TP} remains unchanged : $D_{TP} = D_P$ and by definition of ASD, for every proteins P and Q and for every isometric transformation T we directly have that :

$$\text{ASD}(P, T\,Q) = \text{ASD}(P, Q) \tag{8}$$

1.3 Euclidean bound and coherence with $RMSD_d$

Let us formally state the theoretical bound of ASD values with respect to $RMSD_d$. Indeed, if two fragments are similar in the $RMSD_d$ sense, they will be considered as similar by ASD.

Let P, Q be two protein fragments that are thought to be similar (meaning that one can align the residues one-to-one). We define $\text{RMSD}_\text{d}(P, Q)$ as :
$\text{RMSD}_\text{d}(P, Q) := \sqrt{\frac{1}{\binom{N}{2}} \sum_{i<j<n} (D_{P\,i,j} - D_{Q\,i,j})^2}$

We can bound ASD by $\text{RMSD}_\text{d}(P, Q)$:

$$\begin{aligned} \text{ASD}(P, Q) &= ||\,|\mathcal{F}D_P| - |\mathcal{F}D_Q|\,||_2 \\ &\leq ||\,\mathcal{F}D_P - \mathcal{F}D_Q\,||_2 \\ &\leq ||\,D_P - D_Q\,||_2 \\ &\leq \sqrt{\binom{N}{2}}\, RMSD_\text{d}(P, Q) \end{aligned} \tag{9}$$

In order to theoretically compare ASD with classical $RMSD$, we must suppose that the difference between two structures lies in only small deformations, as shown in the next section.

1.4 Small sensitivity to small changes

We prove here the fact that ASD is a *gradual* dissimilarity: a deformation of a fragment will end with at worst a proportional (i.e. linear) change of the value of ASD. Moreover, we find a common bound to $RMSD$ and ASD in case of local variations.

Formally, a "small" change of a fragment can be captured by a function $f : \mathbb{R}^3 \longrightarrow \mathbb{R}^3$ such that $\forall x \in \mathbb{R}^3, ||x - f(x)||_2 \leq \epsilon$. Then for any such f:

$$\begin{aligned} \mathrm{RMSD}(P, f\,P) &= \sqrt{\frac{1}{N} \sum_{0 \leq i \leq N-1} ||x_i - f(x_i)||_2^2} \\ &\leq \sqrt{\frac{1}{N} \sum_{0 \leq i \leq N-1} \epsilon^2} \\ &\leq \sqrt{\frac{1}{N} N \epsilon^2} \\ &\leq \epsilon \end{aligned} \tag{10}$$

And on the other hand $\forall x, y \in \mathbb{R}^3$:

$$\begin{aligned} ||f(x) - f(y)||_2 &= ||f(x) - x + x - y + y - f(y)||_2 \\ &\leq ||f(x) - x||_2 + ||x - y||_2 + ||y - f(y)||_2 \\ &\leq ||x - y||_2 + 2 \times \epsilon \end{aligned} \tag{11}$$

And similarly : $\forall x, y \in \mathbb{R}^3 ||x - y||_2 \leq ||f(x) - f(y)||_2 + \epsilon$

So that: $||D_P - D_{f\,P}||_2 \leq 2N\epsilon$

So that using Euclidean bound property (cf. section 1.3):

$$\mathrm{ASD}(P, f(P)) \leq 2N\epsilon \tag{12}$$

So that in case of small variation of structure, ASD and $2N \times RMSD$ are bounded by the same $2N\epsilon$.

And using triangle inequality of (cf. section 1.7), for arbitrary proteins P, Q and arbitrary ϵ-quasi-isometry f we obtain:

$$\mathrm{ASD}(P, Q) - 2N\epsilon \leq \mathrm{ASD}(f(P), Q) \leq \mathrm{ASD}(P, Q) + 2N\epsilon \tag{13}$$

This means that *applying small deformations over a protein structure will result at most into a proportional change of ASD.*

1.5 Invariance by circular permutation

We prove here a technical result about ASD: the value of ASD is invariant by circular permutation of the sequence of the residues in the fragment. This result is crucial to formally prove later when dealing with the padded version $\widetilde{\mathrm{ASD}}$ that for two fragments P and Q, one has $\widetilde{\mathrm{ASD}}(P, Q) \leq ||D_{P \setminus Q}||_2$ where $D_{P \setminus Q}$ is the difference of the distance matrices in *the optimal shift* of matrices, meaning that, at most, $\widetilde{\mathrm{ASD}}$ measures *only where P and Q differ.*

Let us consider the simpler case of a N-dimensional vector $\vec{x} := (x_0, ..., x_{N-1})$ of $\mathbb{R}^N$. If we define a circularly permuted version $\vec{y}$ of the previous vector by $y_k := x_{k-s}$,

then:

$$
\begin{aligned}
\mathcal{F}\vec{y}_n &= \frac{1}{\sqrt{N}} \sum_k y_k . e^{-2i\pi kn/N} \\
&= \frac{1}{\sqrt{N}} \sum_k x_{k-s} . e^{-2i\pi kn/N} \\
&= \frac{1}{\sqrt{N}} \sum_k x_k . e^{-2i\pi (k+s)n/N} \\
&= \frac{1}{\sqrt{N}} \sum_k x_k . e^{-2i\pi kn/N} . e^{-2i\pi sn/N} \\
&= e^{-2i\pi sn/N} . \frac{1}{\sqrt{N}} \sum_k x_k . e^{-2i\pi kn/N} \\
&= e^{-2i\pi sn/N} . \mathcal{F}\vec{x}_n
\end{aligned} \tag{14}
$$

Hence,

$$
\forall n, |\mathcal{F}\vec{y}_n| = |\mathcal{F}\vec{x}_n| \tag{15}
$$

Similarly, in dimension 2, if we denote by $M \gg s$ the circularly permuted version of shift s of the matrix M, i.e. $(M \gg s)_{i,j} := M_{i+s,j+s'}$, then one has:

$$
|\mathcal{F}M| = |\mathcal{F}(M \gg s)| \tag{16}
$$

Thus, by defining $P \gg s$ to be the protein whose distance matrix is $D_{(P \gg s)} := D_P \gg s$, we get:

$$
\mathrm{ASD}(P, P \gg s) = 0 \tag{17}
$$

1.6 Invariance by sequential inversion

As in the previous section let us consider the simpler case of a N-dimensional vector $\vec{x} := (x_0, ..., x_{N-1})$ of $\mathbb{R}^N$. We denote the sequential inversion of vector $\vec{x}$ by $\overleftarrow{x} := (x_{N-1}, ..., x_0)$, i.e. $\overleftarrow{x}_i = \vec{x}_{N-1-i}$. Then,

$$
\begin{aligned}
\mathcal{F}\overleftarrow{x}_n &= \sum_k x_{N-k} . e^{-2i\pi kn/N} \\
&= \frac{1}{\sqrt{N}} \sum_k x_k . e^{-2i\pi (N-k)n/N} \\
&= \frac{1}{\sqrt{N}} \sum_k x_k . e^{-2i\pi N/N} . e^{2i\pi kn/N} \\
&= \frac{1}{\sqrt{N}} \sum_k x_k . e^{2i\pi kn/N} \\
&= \overline{\mathcal{F}\vec{x}_n}
\end{aligned} \tag{18}
$$

Hence,

$$
\forall n, |\mathcal{F}\overleftarrow{x}_n| = |\mathcal{F}\vec{x}_n| \tag{19}
$$

Similarly, for the 2-dimensional case, if M' is a sequentially inverted version of a matrix M, i.e. $M'_{i,j} := M_{N-1-i,N-1-j}$, then one has:

$$
|\mathcal{F}M'| = |\mathcal{F}M| \tag{20}
$$

The distance matrix $D_{\overline{P}}$ of a sequentially inverted protein P is the sequential inversion of matrix D_P. By the previous equation, we get:

$$\text{ASD}(\overline{P}, P) = 0 \tag{21}$$

And for arbitrary proteins P, Q:

$$\text{ASD}(\overline{P}, Q) = \text{ASD}(P, Q) \tag{22}$$

1.7 ASD is a pseudometric

To prove that ASD is a pseudometric, let us first prove that ASD respect triangle inequality.

For three arbitrary proteins P,Q,R, one has:

$$\begin{aligned} \text{ASD}(P, R) &= \| \, |\mathcal{F}D_P| - |\mathcal{F}D_R| \, \|_2 \\ &= \| \, |\mathcal{F}D_P| - |\mathcal{F}D_Q| + \\ &\qquad |\mathcal{F}D_Q| - |\mathcal{F}D_R| \, \|_2 \\ &\leq \| \, |\mathcal{F}D_P| - |\mathcal{F}D_Q| \, \|_2 + \\ &\qquad \| \, |\mathcal{F}D_Q| - |\mathcal{F}D_R| \, \|_2 \\ &\leq \text{ASD}(P, Q) + \text{ASD}(Q, R) \end{aligned} \tag{23}$$

Up to here, we can state the following properties:

- $\forall P, \text{ASD}(P, P) = 0$
- $\forall P, Q, \text{ASD}(P, Q) = \text{ASD}(Q, P)$
- $\forall P, Q, R, \text{ASD}(P, R) \leq \text{ASD}(P, Q) + \text{ASD}(Q, R)$

Thus, ASD is a pseudometric. However ASD is not totally a metric over the fragments because we can have

$$ASD(P, Q) = 0 \wedge P \neq Q. \tag{24}$$

By taking for example $Q := \overline{P}$ and applying property of invariance by sequential inversion of 1.6 (let us remark that in that case, $\text{RMSD}(P, Q) \neq 0$).

2 ASD variants

2.1 Padded ASD

If two fragments are superimposable over a subpart of them, we show here that a slight variant of ASD, denoted $\widetilde{\text{ASD}}$, will be lower than the difference of the distance matrices out of this subpart. That is to say that $\widetilde{\text{ASD}}$ will measure at most the difference between the two fragment only where they differ.

First, suppose we have two protein fragments P and Q that superimpose exactly over a subpart of them. Formally speaking, there exists a, b and a shift s such that ${D_P}_{i,j} = {D_Q}_{i+s,j+s}$ for $a \leq i, j \leq b$.

We denote by $D_Q \gg s$ the circularly permuted version of shift s of D_Q:

$$(D_Q \gg s)_{i,j} := {D_Q}_{i+s,j+s} \tag{25}$$

Using the properties of circular permutation of 1.5 and Euclidean bound of 1.3, we see that:

$$\begin{aligned} \mathrm{ASD}(P,Q) &:= || \, |\mathcal{F}D_P| - |\mathcal{F}D_Q| \, ||_2 \\ &= || \, |\mathcal{F}D_P| - |\mathcal{F}(D_Q \gg s)| \, ||_2 \\ &\leq || \, D_P - D_Q \gg s \, ||_2 \end{aligned} \tag{26}$$

And $D_P - D_Q \gg s$ is zero where P and Q superimpose. Thus, ASD measures at worst the difference between P and Q where they disagree. But this measure can be meaningless since by circularly permuting D_Q one compares unrelated parts of fragments (i.e. the beginning of fragment P with the end of fragment Q and vice versa). In order to and with a more meaningful upper bound than in equation 26, we now introduce a padded variant of ASD.

Consider now that we pad the distance matrices D_P of dimension $N_P \times N_P$ and D_Q of dimension $N_Q \times N_Q$ of protein fragments P and Q into bigger matrices $\widetilde{D_P}$ and $\widetilde{D_Q}$ of dimension $N \times N$ where $N = N_P + N_Q$. The padding is done with zeros.

As in equation 26, we get:

$$\widetilde{\mathrm{ASD}}(P,Q) \leq || \, \widetilde{D_P} - \widetilde{D_Q} \gg s \, ||_2 \tag{27}$$

But $\widetilde{D_P} - \widetilde{D_Q} \gg s$ is non-zero only where P and Q differ: out of the rectangle defined by a, b. Taking s to be the shift obtain in the optimal superimposition, we end with:

$$\widetilde{\mathrm{ASD}}(P,Q) \leq ||D_{P \setminus Q}||_2 \tag{28}$$

where $D_{P \setminus Q}$ is the difference of the distance matrices in the optimal alignment of the matrices (i.e. minimizing $RMSD_d$).

That is to say $\widetilde{ASD}$ measures at worst only the difference between P and Q only where P and Q differ.

Author details

References

1. Jain, A.K.: Fundamentals of Digital Image Processing. Prentice-Hall, Inc., Upper Saddle River, NJ, USA (1989)
